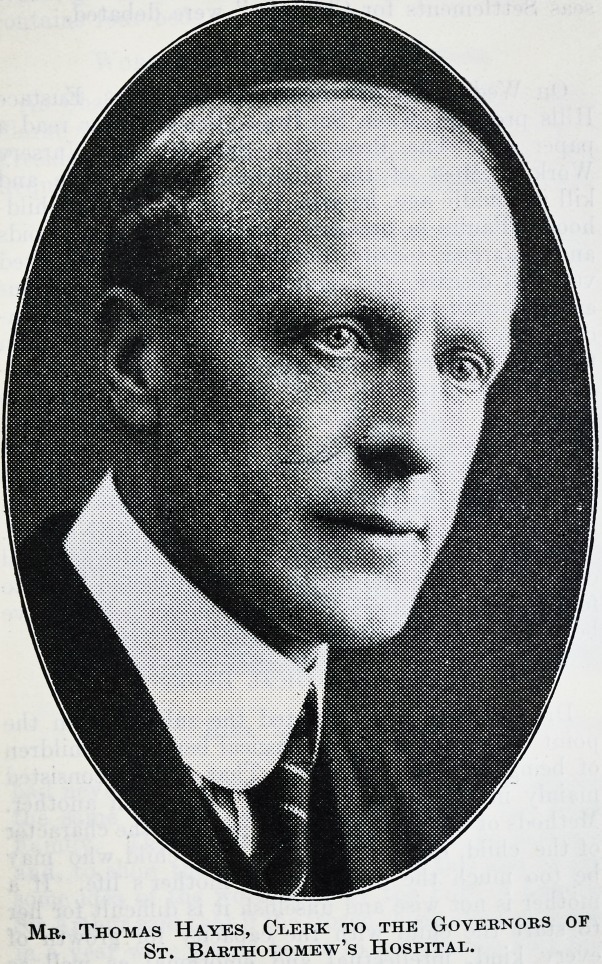# Hospital Men of Mark: Mr. Thomas Hayes

**Published:** 1924-08

**Authors:** 


### HOSPITAL MEN OF MARK.

### MR. THOMAS HAYES.

This year Mr. Thomas Hayes, Clerk to the Governers of St. Bartholomew's Hospital, completes thirty years of hospital service, nearly twenty of which have been passed in his present post. Indeed, it is remarkable that he has held only two hospital secretaryships, both in London, and has, therefore, devoted the best part of his active life to the East London Hospital for Children, Shadwell, and to

Mr. Thomas Hayes, Clerk to the Governors of St. Bartholomew's Hospital.

St. Bartholomew's. It was in 1894 that Mr. Hayes went to Shadwell, which was founded in 1868, and he remained there till February, 1905, when be became chief administrative officer at " Barts." The post carries with it the representation in all departments of the treasurer and almoners in their absence, and during Mr. Hayes's tenure he has served under three treasurers, Lord Ludlow, Lord Sandhurst, and Lord Stanmore.

Probably important changes succeed each other more rapidly in the great hospitals than in the smaller, and St. Bartholomew's has been no exception to this rule. Since 1905 the alterations and extensions include the building of a new out-patients' block and resident staff quarters, a pathological block, and the first wing of a new Home for the nurses. Within the same period the nursing staff has increased from 237 to 323. The new wing was lately completed and is now occupied, and the building of a further wing is about to be begun. But the gradual reconstruction of St. Bartholomew's is now being considered, and Mr. Hayes is honorary secretary to a small special committee which has been entrusted with the task of preparing a scheme and plans whereby the reconstruction may be gradually carried out.

An event that will always mark the past year in the hospital's history was the celebration of its octocentenary, and the general public no less than hospital men have lively recollections of the elaborate functions which were held on that occasion. It is possible that Mr. Hayes found the work entailed behind the scenes last year as arduous as any that has fallen to him in connection with the hospital's growth during his tenure of office. But some of the larger general problems of the voluntary system have also engaged his attention. He gave evidence before Lord Cave's Committee of Inquiry and on the question of State assistance to hospitals and the possibility of supplementing their income when the post-war crisis in hospital finance was causing alarm. As the representative deputed by the Governors to give evidence on this matter, Mr. Hayes advocated the principle of payment for services rendered in the treatment of all classes of patients for which the Government or local authorities were under statutory obligation to provide.

Mr. Hayes has been a member of the Council of the British Hospitals Association since 1910, when the old Hospitals Association was refounded as the British Hospitals Association. He was a member of the Executive Committee and frequently served on deputations during the negotiations between the Association and various Government departments in regard to the treatment of wounded soldiers during the war and of pensioners afterwards. In 1919 the British Hospitals Association took the important decision to form Regional Committees for the better co-ordination of the Association's work. A new method of election to the Council was instituted, and the Council now consists almost entirely of representatives elected by the Regional Committees. A few seats can be provided for the chief personalities of the hospital world. The advantage of the change is that the Council is now really representative of the considered opinion of hospitals throughout the country ; and there is no doubt that not the Council only, but the British Hospitals Association as a whole has been enormously strengthened since the Regional Committees were formed. It was found necessary to revise the rules, and those under which the British Hospitals Association is now administered were drafted by Mr. Hayes in 1920, and for this important work he received the Association's thanks. Mr. Hayes was also one of the first members of the Library Committee of the Association that was appointed in 1920. He continues to serve in that capacity and, generally, to take the greatest interest in furthering the scope and usefulness of the work of the British Hospitals Association.

## Figures and Tables

**Figure f1:**